# Nationale Umfrage zur Präsenz von interdisziplinären Fallkonferenzen bei interstitiellen Lungenerkrankungen (ILD-Boards) an Kliniken in Deutschland

**DOI:** 10.1007/s00393-025-01660-w

**Published:** 2025-08-07

**Authors:** Claus-Jürgen Bauer, Dirk Skowasch, Michael Kreuter, Okka W. Hamer, Jürgen Behr, Sven Gläser, Claus Peter Heussel, Daniel Kütting, Andreas Krause, Gabriela Leuschner, Philipp Markart, Simon Michael Petzinna, Markus Polke, Valentin Sebastian Schäfer

**Affiliations:** 1 Medizinische Klinik III für Onkologie, Hämatologie, Zell- und Immuntherapien, Klinische Immunologie und Rheumatologie, Universitätsklinikum Bonn, Bonn, Deutschland; 2 Medizinische Klinik und Poliklinik II, Sektion Pneumologie, Universitätsklinikum Bonn, Bonn, Deutschland; 3 Lungenzentrum Mainz, Klinik für Pneumologie, Beatmungs- und Schlafmedizin, Marienhaus Klinikum Mainz und Klinik für Pneumologie, Universitätsmedizin Mainz, Mainz, Deutschland; 4 Institut für Röntgendiagnostik, Universitätsklinik Regensburg, Regensburg, Deutschland; 5 Medizinische Klinik und Poliklinik V, LMU Klinikum der Universität München, München, Deutschland; 6 Deutsches Zentrum für Lungenforschung, Gießen, Deutschland; 7 Vivantes Klinikum Spandau und Neukölln, Berlin, Deutschland; 8 Klinik für Diagnostische und Interventionelle Radiologie, Universitätsklinikum Heidelberg, Heidelberg, Deutschland; 9 Translational Lung Research Center (TLRC), Deutsches Zentrum für Lungenforschung (DZL), Heidelberg, Deutschland; 10 Diagnostische und interventionelle Radiologie mit Nuklearmedizin, Thoraxklinik, Universitätsklinikum Heidelberg, Heidelberg, Deutschland; 11 Klinik für Diagnostische und Interventionelle Radiologie, Universitätsklinikum Bonn, Bonn, Deutschland; 12 Abteilung Rheumatologie, Klinische Immunologie und Osteologie, Immanuel Krankenhaus Berlin, Berlin, Deutschland; 13 Medizinische Klinik 5 (Pneumologie), Klinikum Fulda gAG, Campus Fulda, Universitätsmedizin Marburg, Fulda, Deutschland; 14 Zentrum für interstitielle und seltene Lungenerkrankungen, Pneumologie und Beatmungsmedizin, Thoraxklinik, Universitätsklinikum Heidelberg, Heidelberg, Deutschland; 15 Universitätsklinikum Gießen und Marburg, Standort Gießen, Gießen, Deutschland

**Keywords:** ILD-Board, Interstitielle Lungenerkrankungen, Interdisziplinäre Fallkonferenz, Rheumatologie, Pneumologie, Radiologie, ILD-MDM, Lung diseases, interstitial, Interdisciplinary case conference, Rheumatology, Pulmonology, Radiology

## Abstract

**Hintergrund:**

Interstitielle Lungenerkrankungen (ILD) stellen eine interdisziplinäre klinische Herausforderung dar und sind nicht selten mit rheumatologischen Erkrankungen assoziiert. ILD-Boards bieten eine strukturierte Plattform zur interdisziplinären Fallbesprechung und Entscheidungsfindung. Trotz ihrer hohen Bedeutung in der Patientenversorgung fehlt es an Daten zur Verbreitung, Struktur und Arbeitsweise von ILD-Boards in Deutschland.

**Ziel der Arbeit:**

Ziel der Studie war die Erfassung des Status quo von ILD-Boards an deutschen Kliniken sowie deren Zusammensetzung, Abläufe und Optimierungspotenziale.

**Material und Methoden:**

Es erfolgte eine webbasierte Umfrage via SurveyMonkey unter der Schirmherrschaft der Deutschen Gesellschaft für Rheumatologie und Klinische Immunologie (DGRh) und in Zusammenarbeit mit der Deutschen Gesellschaft für Pneumologie und Beatmungsmedizin (DGP) sowie der Deutschen Röntgengesellschaft (DRG). Ein standardisierter Fragebogen erfasste Informationen zu beteiligten Fachdisziplinen, organisatorischen Strukturen sowie Inhalten und Herausforderungen der lokalen ILD-Boards. Die Auswertung erfolgte deskriptiv.

**Ergebnisse:**

An der Studie nahmen insgesamt 125 Ärzte aus 15 Bundesländern teil. Pneumologen (93,6 %), Radiologen (86,4 %), Rheumatologen (59,2 %) und Pathologen (57,6 %) bilden die häufigsten Mitglieder von ILD-Boards. Mehrheitlich finden die erfassten ILD-Boards in Präsenz (50 %) oder hybrid (31,5 %) und 1‑mal wöchentlich (41,1 %) statt. Zwei Drittel der besprochenen Patientenfälle erhalten eine definitive Diagnosestellung und Therapieempfehlung.

**Diskussion:**

Die Ergebnisse zeigen eine hohe Akzeptanz und Verbreitung von ILD-Boards in Deutschland, jedoch auch Optimierungspotenziale, insbesondere in Bezug auf die interdisziplinäre Beteiligung, technische Infrastruktur und Standardisierung.

**Zusatzmaterial online:**

Die Online-Version dieses Beitrags (10.1007/s00393-025-01660-w) enthält die Liste der deutschsprachigen Fragen.

## Abstract (en)

**Background:**

Interstitial lung diseases (ILD) represent an interdisciplinary clinical challenge and are not uncommonly associated with rheumatological diseases. Interstitial lung disease multidisciplinary meetings (ILD-MDM) provide a structured platform for interdisciplinary case discussions and decision making. Despite their great importance in patient care, data on the prevalence, structure and function of ILD-MDM in Germany are lacking.

**Objective:**

The aim of the study was to assess the current status of ILD-MDM in German hospitals to gain insights into their composition, processes and potential for optimization.

**Material and methods:**

A web-based survey was conducted via SurveyMonkey under the auspices of the German Society for Rheumatology and Clinical Immunology (DGRh) and in collaboration with the German Respiratory Society (DGP) and the German Radiological Society (DRG). A standardized questionnaire captured information on the participating specialist disciplines, organizational structures as well as the content and challenges of local ILD-MDM. The analysis was conducted descriptively.

**Results:**

A total of 125 physicians from 15 federal states in Germany participated. Pulmonologists (93.6%), radiologists (86.4%), rheumatologists (59.2%) and pathologists (57.6%) are the most commonly represented members of ILD-MDM. The majority of ILD-MDMs are conducted either in person (50%) or in a hybrid format (31.5%) and are held on a weekly basis (41.1%). Of all patient cases discussed, two thirds receive a definitive diagnosis and treatment recommendation.

**Conclusion:**

The findings reveal a high acceptance and prevalence of ILD-MDM in Germany but also highlight potential areas for improvement, particularly regarding interdisciplinary participation, technical infrastructure and standardization.

## Hintergrund und Fragestellung

Interstitielle Lungenerkrankungen („interstitial lung diseases“ [ILD]) stellen eine bedeutende klinische Herausforderung in der radiologischen Befundung sowie pneumologischen und rheumatologischen Patientenversorgung dar.

Verschiedene rheumatologische Erkrankungen (darunter führend die systemische Sklerose, die rheumatoide Arthritis sowie die Myositiden) können mit einer ILD einhergehen und hierdurch prognostisch ungünstig beeinträchtigt werden [1]. Außerdem kann die ILD als pulmonale Manifestation in etwa 10 % der Fälle einer späteren rheumatologischen Systemerkrankung vorausgehen [2]. Daneben können ILDs sowohl auf dem Boden anderer fassbarer Ursachen (beispielsweise als granulomatöse Erkrankungen wie die Sarkoidose, ausgelöst durch inhalative Noxen/Allergene wie die exogen-allergische Alveolitis oder medikamentös induziert) entstehen als auch idiopathisch, d. h. ohne erkennbare Ursache auftreten. In Fortführung des bereits zwischen 1990 und 2017 beobachtbaren Anstiegs an ILD-mortalitätsvermittelt verlorenen Lebensjahren um 86 %, ist in den kommenden 2 Dekaden mit einer weiteren Verdopplung zu rechnen [3].

Wie bereits 2002 erstmalig in einem gemeinsamen Statement der American Thoracic Society und European Respiratory Society empfohlen, erfordert die Komplexität der Diagnostik und Therapie einer ILD eine enge interdisziplinäre Zusammenarbeit zwischen Pneumologie, Rheumatologie, Radiologie und Pathologie sowie bedarfsgerecht weiteren benötigten Fachdisziplinen [4, 5]. In diesem Kontext haben sich Fallbesprechungen zu interstitiellen Lungenerkrankungen (ILD-Boards) als wertvolles Instrument und Goldstandard etabliert, um eine koordinierte, interdisziplinär fundierte Entscheidungsfindung zu gewährleisten [6, 7] und die Patientenversorgung zu optimieren.

ILD-Boards bieten die Möglichkeit, Erkrankungsfälle strukturiert zu diskutieren, bildgebende, histopathologische, klinische sowie Laborbefunde zu demonstrieren, im interdisziplinären Austausch die Diagnosefindung zu bewerkstelligen und personalisierte Therapiepläne zu entwickeln. Trotz der wachsenden Bedeutung solcher Boards in der klinischen Praxis ist wenig über deren Verbreitung, Struktur und Arbeitsweise in Deutschland bekannt. Insbesondere fehlen nationale Daten zu Aspekten wie der Zusammensetzung der ILD-Boards, der Frequenz und Form ihrer Treffen sowie den Herausforderungen, denen sie begegnen. Diese Wissenslücke erschwert es, bewährte Verfahren zu identifizieren und Optimierungspotenziale aufzuzeigen.

Das vorliegende Forschungsprojekt verfolgt daher das Ziel, den aktuellen Status von ILD-Boards an deutschen Kliniken systematisch zu erfassen.

## Studiendesign und Untersuchungsmethoden

Unter der Schirmherrschaft der Deutschen Gesellschaft für Rheumatologie und Klinische Immunologie (DGRh) und in Zusammenarbeit mit der Deutschen Gesellschaft für Pneumologie und Beatmungsmedizin (DGP) sowie der Deutschen Röntgengesellschaft (DRG) wurde im Rahmen des DGRh-Arbeitskreises „Lungenbeteiligung bei rheumatologischen Erkrankungen“ (Leitung: Univ.-Prof. Dr. med. MUDr. Valentin S. Schäfer, Universitätsklinikum Bonn) unter Einbeziehung eines pneumologischen und radiologischen Experten-Boards in 2 Online-Sitzungen der Datenerhebungsinhalt dieser Studie ausgearbeitet und diskutiert. Im Ergebnis entstand ein Fragenkatalog, bestehend aus 26 Einzelfragen. Die Liste der deutschsprachigen Fragen ist im elektronischen Zusatzmaterial dargestellt. Die vorliegende Studie wurde von der Ethikkommission an der Medizinischen Fakultät Bonn (IRB-No. 288/23) positiv votiert.

Realisiert wurde die webbasierte Befragung mittels SurveyMonkey® (SurveyMonkey Inc., San Mateo, CA, USA). Eine Einladung aller Fachgesellschaftsmitglieder erfolgte über die jeweiligen E‑Mail-Verteiler und Newsletter der DGRh, DGP und DRG. Eine Teilnahme war zwischen dem 10.05.2024 und 31.12.2024 möglich. Doppelnennungen wurden via Adress- und Postleitzahlendaten ausgeschlossen. Nach Abschluss der Datenvollständigkeitsprüfung erfolgte die statistische Auswertung mittels deskriptiver Statistikmethoden über die SurveyMonkey®-eigenen Werkzeuge sowie Microsoft Excel (Version 2411; Microsoft Corp. Redmond, WA, USA).

## Ergebnisse

### Demografie

**Abb. 1:**
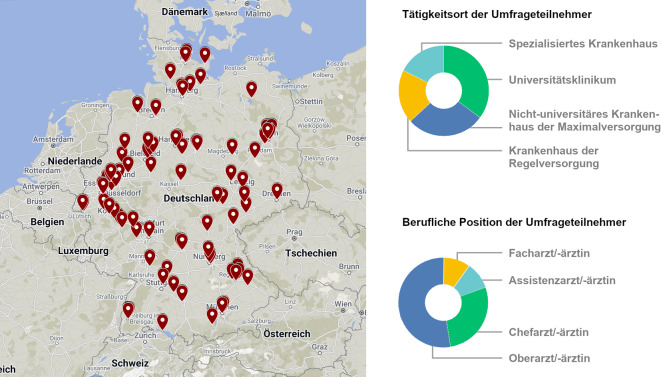
Umfrageteilnehmer-Demografie. Geografische Standortdarstellung nach Postleitzahlabfrage, Einrichtungsart und berufliche Demografie der Umfrageteilnehmenden. Kartenmaterial zugrunde liegend von Google Maps (Stand: Januar 2025). (© Google LLC, Mountain View, CA, USA. Grafische Adaptation: Dr. Claus-Jürgen Bauer)

Insgesamt beantworteten 125 Teilnehmende die Onlineumfrage. Die Abschlussquote betrug 100 %. Der durchschnittliche Zeitaufwand belief sich auf 6 min 8 s (Standardabweichung ±37 min 59 s, bzw. nach Bereinigung von 7 Ausreißern mit > 30 min Bearbeitungszeit, welche auf eine zwischenzeitliche Bearbeitungspause schließen lassen: ±4 min 12 s). Bei den Teilnehmenden handelte es sich zu 28 % um Chefärzte, 52,8 % Oberärzte, 9,6 % Fachärzte und 9,6 % Assistenzärzte. Insgesamt nahmen Rheumatologen, Pneumologen und Radiologen aus 15 Bundesländern teil, namentlich aus Nordrhein-Westfalen (24,0 %), Bayern (15,7 %), Berlin (11,6 %), Baden-Württemberg (10,7 %), Schleswig-Holstein (7,4 %), Niedersachen (6,6 %), Rheinland-Pfalz (5,8 %), Sachsen (4,1 %), Thüringen (4,1 %), Hamburg (2,5 %), Hessen, Bremen, Mecklenburg-Vorpommern und Sachsen-Anhalt (jeweils 1,7 %) sowie Brandenburg (0,8 %). Nicht vertreten zeigte sich das Saarland. Vier Teilnehmende enthielten sich der Angabe ihrer geografischen Herkunft. Die Standorte der Teilnehmenden sind in Abb. 1 kartografiert. Ein Großteil der Teilnehmenden (35,2 %) gab an, derzeit an einem Universitätsklinikum tätig zu sein. Weitere 28,0 % arbeiten an einem nichtuniversitären Krankenhaus der Maximalversorgung, 19,2 % an einem Krankenhaus der Regelversorgung und 17,6 % an einem spezialisierten Krankenhaus. In näherer Spezifizierung der Angabe „Beschäftigung in einem spezialisierten Krankenhaus“ gaben 17 Teilnehmende die Arbeit in einer pneumologisch spezialisierten Fachklinik, 2 Teilnehmende in einer rheumatologisch spezialisierten Fachklinik, 1 Teilnehmender in einer rheumatologisch und pneumologisch spezialisierten Fachklinik und 2 Teilnehmende in einer „Spezialpraxis“ ohne nähere Angabe an.

### Fachliche Zusammensetzung des lokalen ILD-Boards

**Abb. 2:**
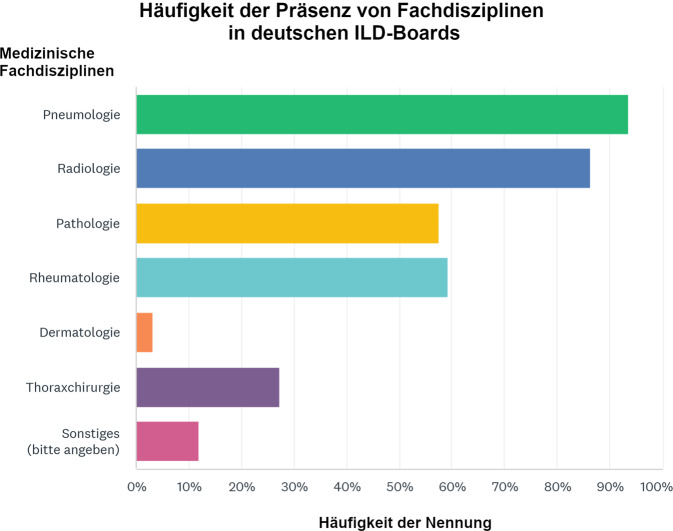
ILD-Board-Fachbesetzung. Frequenz der Nennung verschiedener Fachdisziplinen als vertretene Beteiligte im lokalen ILD-Board. Unter „Sonstiges“ gaben 3 Teilnehmende die Präsenz der Arbeitsmedizin (2,4 %), 2 Teilnehmende die regelhafte Präsenz der Kardiologie (1,6 %), 2 Teilnehmende die Präsenz der Nuklearmedizin (1,6 %) und ein Teilnehmender die regelhafte Repräsentanz der Transplantationsmedizin (0,8 %) an

Als vertretene Fachdisziplinen im lokalen ILD-Board wurden Pneumologie (93,6 %), Radiologie (86,4 %), Rheumatologie (59,2 %) und Pathologie (57,6 %) am häufigsten genannt. Weitere Antwortfrequenzen sind Abb. 2 zu entnehmen. Unter „Sonstiges“ gaben 3 Teilnehmende die Präsenz der Arbeitsmedizin (2,4 %), 2 Teilnehmende die regelhafte Präsenz der Kardiologie (1,6 %), 2 Teilnehmende die Präsenz der Nuklearmedizin (1,6 %) und ein Teilnehmender die regelhafte Repräsentanz der Transplantationsmedizin (0,8 %) an. Darüber hinaus erging von 7 Teilnehmenden die Rückmeldung, dass einzelne Fachrichtungen nur bedarfsweise hinzugezogen würden (konkretisierte Spezifizierung: Pathologie *n* = 1, Rheumatologie *n* = 2, Thoraxchirurgie *n* = 1).

Unter allen Umfrageteilnehmenden gaben 74,2 % zudem an, dass eine Mindestpräsenz von Fachdisziplinen für das Zustandekommen des ILD-Boards an der eigenen Klinik erforderlich ist. In 29 von 92 Fällen (31,5 %) war dies eine Kombination aus Pneumologie und Radiologie, in weiteren 26 von 92 Fällen (28,2 %) unter Einbezug der Rheumatologie bzw. in weiteren 18 von 92 Fällen (19,6 %) Pneumologie und Radiologie unter Einbezug der Pathologie; 7 von 92 Teilnehmenden (7,6 %) gaben die Mindestpräsenz der Konstellation aus Pneumologie, Radiologie, Rheumatologie und Pathologie an. In 5 von 92 Fällen (5,4 %) bestand die Mindestzusammensetzung aus Pneumologie, Radiologie, Pathologie und Thoraxchirurgie, in einem weiteren Fall (1,1 %) unter zusätzlicher Präsenz der Allergologie sowie in einem anderen weiteren Fall (1,1 %) unter zusätzlicher Gegenwart von Nuklearmedizin und Rheumatologie. Unter Verzicht auf den Fachbereich Pathologie wurde in jeweils 2 von 92 Fällen (2,2 %) die erforderliche Mindestpräsenz von Pneumologie, Radiologie und Thoraxchirurgie bzw. von Pneumologie, Radiologie, Thoraxchirurgie und Rheumatologie angegeben. In einem Fall wurde eine Mindestbesetzung ohne Radiologie, ausschließlich durch Pneumologie und Rheumatologie angegeben. Anders ausgedrückt sind unter allen ILD-Boards, die eine Mindestteilnahme bestimmter Fachrichtungen festgelegt haben, einzelne Fachbereiche in nachfolgend genannter Häufigkeit zwingend vertreten: Pneumologie (100 %), Radiologie (98,9 %), Rheumatologie (40,2 %), Pathologie (34,8 %), Thoraxchirurgie (12,0 %), Nuklearmedizin (1,1 %), Allergologie (1,1 %).

Unter allen 125 Umfrageteilnehmenden gaben 37 darüber hinaus an, dass der Fachbereich Pneumologie im eigenen ILD-Board zudem durch mindestens 2 Fachvertreter repräsentiert sein muss. Einmalig erfolgte des Weiteren die Angabe, dass sowohl Pneumologie als auch Rheumatologie durch mindestens 2 Fachvertreter repräsentiert sein müssen.

### Räumliche und zeitliche Konzeption von ILD-Boards

Die mittels der Umfrage deutschlandweit erfassten ILD-Boards finden zum größten Teil ausschließlich in Präsenz (50 %) oder hybrid – also parallel in Präsenz und virtuell – statt (31,45 %). In 18,55 % der Fälle erfolgt die Durchführung ausschließlich virtuell. Am häufigsten ist dabei ein wöchentliches Durchführungsintervall (41,1 %), gefolgt von einer 1‑mal monatlichen (25,0 %) oder 2‑mal monatlichen Durchführung (17,7 %). Viermal erging die Angabe einer 2‑mal wöchentlichen ILD-Board-Sitzung (3,2 %), in einem Fall (0,8 %) erfolgt das ILD-Board gar 4‑mal pro Woche. In 10,5 % der erfassten ILD-Boards findet die Durchführung unregelmäßig, in Abhängigkeit von der Anzahl der Fälle, statt.

Die Dauer einer durchschnittlichen ILD-Board-Sitzung beläuft sich an den meisten Standorten auf 30–60 min (50,0 %), 60–90 min (24,2 %) oder 15–30 min (18,6 %), seltener auf über 90 min (4,0 %) oder unter 15 min (3,2 %). Als durchschnittlich besprochene Anzahl an Patientenfällen pro ILD-Board-Sitzung wird am häufigsten angegeben „5–10 Fälle“ (37,1 %), „1–5 Fälle“ (30,7 %) oder „10–15 Fälle“ (23,4 %); eine höhere Fallzahl ist deutlich seltener („15–20 Fälle“: 5,7 %; „20–30 Fälle“: 2,4 %; „> 30 Fälle“: 0,8 %). Die Tab. 1 gibt einen Überblick über die Merkmale lokaler ILD-Boards in Deutschland in Gegenüberstellung zu publizierten Daten des europäischen und globalen Vergleichs.

**Tab. 1:** ILD-Board-Merkmale im internationalen Vergleich

ILD-Board-Merkmal	Deutschlandweit (%)	Europaweit (%) (Quelle: [8])	Global (%) (Quelle: [8])
Pneumologie präsent	93,6	n. a.	99,7
Radiologie präsent	86,4	n. a.	91,4
Rheumatologie präsent	59,2	n. a.	37,1
Pathologie präsent	57,6	n. a.	66,3
Zusammenkunft mind. alle 2 Wochen	62,8	60,8	66,9
Zusammenkunft mind. wöchentlich	45,1	n. a.	34,9
Durchführung ausschließlich in Präsenz	50,0	81,1	80,0
Dauer 31–60 min	50,0	48,6	50,9
Besprechung von 1 bis 5 Patientenfällen pro Sitzung	30,7	n. a.	61,0
Besprechung von 5 bis 10 Patientenfällen pro Sitzung	37,1	n. a.	29,0
Besprechung von 10 bis 15 Patientenfällen pro Sitzung	23,4	n. a.	8,0
Besprechung von 15 bis 20 Patientenfällen pro Sitzung	5,7	n. a.	2,0

Gegenüberstellung der Häufigkeit, mit der verschiedene ILD-Board-Charakteristika in ILD-Boards in Deutschland, europaweit und weltweit zutreffen

*n.* *a.* nicht angegeben

### Patientenanmeldung und Zugang zu ILD-Boards

In 84,7 % aller erfassten ILD-Boards besteht die Möglichkeit auch für niedergelassene Kollegen bzw. auswärtige Krankenhäuser, externe Patienten für das ILD-Board anzumelden. Im Durchschnitt aller ILD-Boards stammen 14,7 % (±14,6) der vorgestellten Patientenfälle von externen Anmeldern.

Mehrheitlich (80,5 %) erfolgt die Patientenanmeldung für das ILD-Board über ein standardisiertes Formular. Die darin abgefragten patientenbezogenen Daten umfassen besonders häufig den Raucherstatus (90,3 %), Berufsanamnese, Autoimmundiagnostikergebnisse, Pathologiebefund und Befund der letzten Computertomographie und CT-Muster (jeweils 87,6 %) sowie Medikamentenanamnese (86,7 %), Befunde der bronchoalveolären Lavage (85,8 %), Bodyplethysmographie (84,1 %) und Symptomdauer (81,4 %). Seltener ist eine Angabe des Nachweises spezifischer Immunglobulin-G-Antikörper gegen typische Antigene, die eine exogen-allergische Alveolitis auslösen können (72,6 %), der körperlichen Untersuchungsbefunde (61,1 %) oder des CRP-Wertes (42,5 %) erforderlich.

### Ablauf und inhaltliche Ausgestaltung deutscher ILD-Boards

In lediglich 0,8 % der erfassten ILD-Boards erfolgt die Evaluation radiologischer Befunde ausschließlich durch das Vorlesen des schriftlichen Befundberichts. Hingegen werden in 99,2 % der ILD-Boards die radiologischen Befunde üblicherweise bildlich demonstriert und das ILD-Muster interdisziplinär diskutiert. Die Evaluation der histopathologischen Befunde im Rahmen des ILD-Boards beschränkt sich in 56,5 % der Fälle auf ein Vorlesen des schriftlichen Befundberichts, während in 43,5 % der lokalen ILD-Boards auch eine Bilddemonstration und Diskussion des histopathologischen Befundes erfolgen.

Die überwiegende Mehrheit der Umfrageteilnehmenden (97,6 %) gab an, dass im Rahmen des ILD-Boards an der eigenen Klinik sowohl eine spezifische Diagnosezuordnung erfolgt als auch Therapieempfehlungen ausgesprochen werden. Bei 98,4 % der Befragten umfasst das ILD-Board zudem Empfehlungen zur weiteren Diagnostik. Diesbezüglich gaben 91,9 % der Umfrageteilnehmenden an, dass sie und ihr interdisziplinäres Team in der ILD-Diagnostik typischerweise den Empfehlungen der „S1-Leitlinie Interdisziplinäre Diagnostik interstitieller Lungenerkrankungen im Erwachsenenalter“ [9] folgen würden. Nach Angaben der Umfrageteilnehmenden resultiert die Besprechung eines Patientenfalls in ihrem ILD-Board in 32,6 % (±21,6) der Fälle in der Empfehlung weiterer Diagnostik (z. B. einer Kryobiopsie), sowie in 30,5 % (±22,1) der Fälle in der Empfehlung einer erneuten Computertomographiebildgebung. In 27,5 % (±15,9) der Fälle ergeht die Empfehlung zur ergänzenden rheumatologischen Vorstellung und Mitbeurteilung des Patienten. Zu 62,0 % (±20,1) mündet die Patientenfallbesprechung in einer gesicherten Diagnose. Eine Therapieempfehlung erhalten 69,3 % (±22,7) der besprochenen Patientenfälle. Nach Abschluss der Besprechung eines Patientenfalls erfolgt in etwa zwei Drittel (66,1 %) der erfassten ILD-Boards die Erstellung eines standardisierten Ergebnisprotokolls.

Nicht immer ist ein abschließender Beschluss in einem Patientenfall zum gegenwärtigen Zeitpunkt der ILD-Board-Sitzung möglich. Durchschnittlich muss nach Angaben der Umfrageteilnehmenden in 12,1 % (±12,1) der Fälle die Patientenbesprechung im ILD-Board aufgrund fehlender Befunde verschoben werden. Für solche und ähnliche Situationen finden in 91,9 % der erfassten nationalen ILD-Boards Patientenwiedervorstellungen zur Verlaufsbesprechung statt, beispielsweise nach erfolgter Kryobiopsie oder bei klinischer Verschlechterung.

### Verbesserungsansätze zukünftiger ILD-Boards

Final geschlossen wurde die Onlineumfrage mit der offenen Freitext-Frage: „Wie könnte man zukünftige ILD-Boards weiter verbessern?“ Insgesamt 78 von 125 Umfrageteilnehmenden (62,4 %) machten hierzu Angaben. Die hierbei meistgenannten Anregungen und Wünsche entstammen 4 übergeordneten Themenbereichen. In 20 von 78 Antworten (25,6 %) wurde der Wunsch nach der Teilnahme weiterer Fachdisziplinen formuliert, führend darunter der Rheumatologie (10/78 bzw. 12,8 %) und Pathologie (10/78 bzw. 12,8 %). Für eine Verbesserung der technischen Voraussetzungen zur virtuellen ILD-Board-Teilnahme bzw. die Schaffung solcher Möglichkeiten plädierten 16 von 78 Teilnehmenden (20,5 %), womit dies die zweithäufigste genannte Maßnahme war. Als Hürde wurde hier insbesondere die Wahrung der Datenschutzkonformität angeführt. Ein virtuelles bzw. hybrides Angebot zur ILD-Board-Teilnahme wurde auch wiederholt in Verbindung mit dem Vorschlag einer Öffnung des ILD-Boards für klinikexterne Teilnahmeinteressierte (beispielsweise niedergelassene Fachärzte) genannt (7/78 bzw. 9,0 %).

Am dritthäufigsten (12/78 bzw. 15,4 %) erging der Wunsch nach einem höheren Maß an Standardisierung (insbesondere im Anmeldeprozess und -formular). Die Antwort eines Umfrageteilnehmenden formulierte in diesem Zusammenhang: „Boards sollten nur noch an zertifizierten Zentren zugelassen sein.“

Im vierthäufigst adressierten Themenkomplex (7/78 bzw. 9,0 %) wurden ökonomische Aspekte aufgegriffen. In erster Linie wurde hier eine Verbesserung der Refinanzierung von ILD-Boards genannt, seltener eine konkrete Stärkung personeller und zeitlicher Ressourcen zur Planung, Befundsammlung und Ergebnisdokumentation.

## Diskussion

Die vorliegende Studie bietet eine umfassende nationale Bestandsaufnahme der Struktur, Organisation, inhaltlichen Ausgestaltung und Herausforderungen interdisziplinärer ILD-Boards in Deutschland. Als erste strukturierte Untersuchung dieser Art in Deutschland liefert sie wertvolle Erkenntnisse über die Zusammensetzung, Arbeitsweise und Weiterentwicklungspotenziale dieser zentralen Diskursplattform zur Diagnostik und Therapie interstitieller Lungenerkrankungen.

Die in unseren Studienergebnissen dokumentierte nahezu lückenlose Präsenz der Pneumologie und Radiologie unterstreicht deren zentrale Bedeutung in ILD-Boards und steht im Einklang mit den global erfassten Daten von Richeldi et al. aus dem Jahr 2019 [8]. Die Fachbereiche Rheumatologie und Pathologie stellen deutschlandweit in knapp zwei Drittel der ILD-Boards einen festen Bestandteil dar – gerade die rheumatologische Präsenz liegt damit über der im internationalen Vergleich (vorliegende Umfrage: 59,2 % vs. global: 37,1 % [8]). Grundsätzlich bilden die genannten Fachdisziplinen das Rückgrat der interdisziplinären Diagnostik [4, 5]. Dennoch zeigt die mancherorts begrenzte Beteiligung bzw. Verfügbarkeit der Rheumatologie und Pathologie – insbesondere im Vergleich zu pneumologischen und radiologischen Fachvertretern – einen wichtigen Bereich mit Optimierungspotenzial auf, was auch durch die wiederholt geäußerte Forderung der Studienteilnehmenden nach einer stärkeren Einbindung dieser Fachrichtungen gestützt wird. Hinsichtlich des Fachbereichs Pathologie könnte die teilweise fehlende Involvierung in ILD-Boards auch im Zusammenhang damit stehen, dass mancherorts keine Kryobiopsien erfolgen und sich aufgrund dessen auch keine Histologiebefunde als Diskussionsgrundlage ergeben. Allerdings sollten gerade Zentren mit etabliertem ILD-Board und der damit zum Ausdruck gebrachten Expertise in der Versorgung von Patienten mit ILD als Qualitätsmerkmal auch den Zugang zur Kryobiopsie – heutzutage Biopsieverfahren der ersten Wahl bei verdächtigter fibrosierender ILD [9, 10] – bieten, zumindest in Kooperation. Ebenso betont die S1-Leitlinie „Interdisziplinäre Diagnostik interstitieller Lungenerkrankungen im Erwachsenenalter“: „Obligate Teilnehmer sind […] auf dem Gebiet des ILDs erfahrene Teilnehmende aus der Pneumologie, (Thorax)-Radiologie und (Thorax)-Pathologie (sofern eine Histopathologie vorliegt).“ [9] Gerade in Fällen, die klinisch, anamnestisch und bildmorphologisch keine sichere Zuordnung erlauben, kommt der histopathologischen Beurteilung nicht selten eine entscheidende Rolle zu und kann die Einschätzung von Klinikern und Radiologen richtungsweisend verändern [6]. Frühere Studien haben bereits gezeigt, dass eine erweiterte interdisziplinäre Zusammenarbeit zu besseren diagnostischen und therapeutischen Entscheidungen führen kann [6, 11]. Insbesondere angesichts rheumatologischer Erkrankungen, denen in bis zu 10 % der Fälle eine ILD als Erstmanifestation vorausgehen kann [2], erscheint auch eine engere Einbindung der Rheumatologie von hoher Bedeutung.

Weitere erfasste Charakteristika zur zeitlichen Struktur von ILD-Boards in Deutschland decken sich ebenfalls stark mit europäischen Vergleichswerten aus 2019, wie beispielsweise Frequenz (Anteil an erfassten ILD-Boards, die mindestens alle 2 Wochen stattfinden: 62,8 % in Deutschland vs. 60,8 % europaweit [8]) und Dauer der Zusammenkünfte (Anteil an erfassten ILD-Boards, die 31–60 min dauern: 50,0 % in Deutschland vs. 48,6 % europaweit [8]). Deutlich geringer als noch 2019 europaweit dokumentiert (81,1 %) ist mittlerweile der deutschlandweit berichtete Anteil an ILD-Boards, welche ausschließlich eine Teilnahme in physischer Präsenz anbieten (50,0 %). Auf diese Entwicklung dürfte die zwischenzeitliche COVID-19-Pandemie einen wesentlichen Einfluss genommen haben. Besonders Patienten und Behandler aus ländlichen Regionen ohne alternative Partizipationsmöglichkeit dürften von der in dieser Zeit signifikant verbesserten Digitalisierung und Virtualisierung profitieren. Nichtsdestotrotz betonten zahlreiche Teilnehmenden weiterhin die Notwendigkeit technischer und datenschutzkonformer Lösungen, um eine flächendeckende virtuelle oder hybride Teilnahme an ILD-Boards zu ermöglichen. Dass virtuelle Teilnahmeangebote die Erreichbarkeit und Effizienz interdisziplinärer Zusammenarbeit signifikant verbessern können, konnte am Beispiel virtueller Tumorboards in der Vergangenheit bereits gezeigt werden [12].

Investitionen in digitale Infrastruktur und virtuelle Übertragungskanäle sowie die ILD-Board-Öffnung für klinikexterne Teilnehmende sind Maßnahmen, welche auch an ökonomische Überlegungen geknüpft sind, wie von einigen Umfrageteilnehmenden kritisch angemerkt wird. Allerdings könnten hier die Lungenzentren nach G‑BA ein Vorreiter sein, bei denen die Öffnung ihrer ILD-Boards für andere Teilnehmende in vielen Fällen als besonderes Merkmal hervorgehoben wird. Insbesondere die Refinanzierung dieser zeit- und ressourcenintensiven Besprechungsrunden bedarf einer stärkeren Berücksichtigung in der Gesundheitsökonomie. Hierfür sprechen auch vorangegangene Studienergebnisse, die zeigen konnten, dass interdisziplinäre Fallbesprechungen langfristig kosteneffektiv sein können, indem sie Fehldiagnosen reduzieren und die Behandlungsqualität verbessern [11]. Einen zentralen Ansatzpunkt zur Reduktion des organisatorischen Aufwands sowie Ressourceneinsatzes, aber auch zur Qualitätssicherung bietet die Standardisierung der Anmeldeprozesse und Dokumentation. Der Wunsch nach einem höheren Grad der Standardisierung, insbesondere durch die Nutzung einheitlicher Anmeldeformulare und Zertifizierung von ILD-Boards, wurde in den Freitextantworten der Studienteilnehmenden wiederholt geäußert. In der Tat deckt sich die hier angefragte Verdichtung der Ausgestaltungsdirektive mit der in der verfügbaren wissenschaftlichen Literatur bisher zurückhaltenden Definition von Mindeststandards und Qualitätskriterien für ILD-Boards. Zwar besteht zwischen internationalen Leitlinien eine grundsätzliche Übereinstimmung hinsichtlich einzelner übergeordneter Prinzipien, wie der interdisziplinären Zusammensetzung von ILD-Boards mit fachlicher Mindestpräsenz von Pneumologie, Radiologie, Pathologie sowie fallbasiert auch Rheumatologie; tiefer gehende Festlegungen zu ILD-Board-Standards finden sich hier allerdings kaum [13]. Eine stärkere Standardisierung, insbesondere der für die Patientenfallanmeldung erfassten Daten, könnte erhebliches Potenzial bergen, die derzeit bei durchschnittlich 12 % liegende Rate an Patientenfällen, deren Besprechung aufgrund fehlender Befunde verschoben werden muss, signifikant zu reduzieren. Ein bedeutender Vorstoß gelang hier bereits mit der S1-Leitlinie „Interdisziplinäre Diagnostik interstitieller Lungenerkrankungen im Erwachsenenalter“ [9], mit der Vorschläge für ein standardisiertes ILD-Board-Anmeldeformular und Protokollformular unterbreitet werden. Im Arbeitskreis Lungenbeteiligung bei rheumatologischen Erkrankungen der DGRh werden wir uns den Herausforderungen der Adaption, der Implementierung eines einheitlichen Dokuments und der Validierung dessen Anwendung widmen. Auch könnte die gesteigerte Standardisierung die zukünftige Einbindung von künstlicher Intelligenz, beispielsweise im Anmeldeprozess und der Patientenfallvorbereitung, begünstigen.

Abschließend lässt sich konstatieren, dass die Häufigkeit einer resultierenden Diagnosestellung sowie Therapieempfehlung der Umfrage-erfassten nationalen ILD-Boards die Effektivität interdisziplinärer Fallkonferenzen bei interstitiellen Lungenerkrankungen in der klinischen Entscheidungsfindung unterstreicht. Dies bekräftigt den ohnehin bestehenden Status von ILD-Boards als „Goldstandard“ in der optimierten Versorgung interstitieller Lungenerkrankungen [13, 14].

Eine Stärke der vorliegenden Studie liegt in der umfassenden nationalen Datenerhebung unter Einbindung verschiedener Fachgesellschaften. Die hohe Abschlussquote (100 %) und die breite regionale Abdeckung tragen zur Generalisierbarkeit der Ergebnisse bei.

Dennoch weist die Studie einige Limitationen auf. Die Datenerhebung erfolgte über eine Online-Umfrage, wodurch eine objektive Validierung der Angaben nicht möglich war. Die freiwillige Teilnahme könnte zu einer Überrepräsentation besonders engagierter oder etablierter Zentren geführt haben. Darüber hinaus basiert die Auswertung auf Selbstauskünften der Teilnehmenden, was potenzielle Verzerrungen durch subjektive Wahrnehmungen mit sich bringen kann.

Die vorliegende Studie hebt die Bedeutung interdisziplinärer ILD-Boards in Deutschland hervor und liefert erstmals umfassende Einblicke in deren Struktur und Arbeitsweise in Deutschland. Die Ergebnisse unterstreichen die Notwendigkeit, bestehende Herausforderungen wie die Einbindung zusätzlicher Fachrichtungen, die Digitalisierung und die Standardisierung von Prozessen anzugehen, um die Effektivität dieser Plattformen weiter zu erhöhen und eine Mindestqualität zu gewährleisten. Durch eine stärkere Vernetzung und kontinuierliche Weiterentwicklung könnten ILD-Boards einen noch größeren Beitrag zur Verbesserung der Versorgung von Patienten mit interstitiellen Lungenerkrankungen leisten.

## Fazit für die Praxis

- Interdisziplinäre ILD-Boards haben sich als wertvolles Instrument in der Diagnostik und Therapie interstitieller Lungenerkrankungen etabliert und ermöglichen eine fundierte Entscheidungsfindung.
- Diese Studie liefert erstmalig umfassende Einblicke in die Struktur, Organisation und Herausforderungen von ILD-Boards in Deutschland und zeigt wichtige Optimierungsmöglichkeiten auf.
- Eine stärkere Standardisierung, insbesondere bei der Patientenfallanmeldung, sowie die Förderung hybrider und virtueller Formate könnten die Effizienz und Zugänglichkeit von ILD-Boards erheblich steigern.
- Die stärkere Einbindung der Fachrichtungen Rheumatologie und Pathologie ist essenziell, um die diagnostische und therapeutische Qualität interdisziplinärer Besprechungen weiter zu verbessern.
- Die kontinuierliche Weiterentwicklung von ILD-Boards durch Standardisierung, Digitalisierung und künstliche Intelligenz birgt enormes Potenzial für die zukünftige Verbesserung der Patientenversorgung.

## Data Availability

Auf begründete Anfrage hin können die Daten dieser Forschungsarbeit zur Verfügung gestellt werden.

## Zusatzmaterial online

Liste der deutschsprachigen Fragen
