# Supplementary material for: National survey on the presence of multidisciplinary meetings for interstitial lung diseases (ILD-MDM) in hospitals in Germany. German version
Source: Z Rheumatol. 2025 Aug 7;84(8):671–9. [Article in German] doi: 10.1007/s00393-025-01660-w (PMC12488742; doi:10.1007/s00393-025-01660-w)
Supplement: Supplementary file 1 — Liste der deutschsprachigen Fragen [file 393_2025_1660_MOESM1_ESM.pdf]

Nationale Umfrage zur Präsenz von interdisziplinären Fallkonferenzen bei interstitiellen Lungenerkrankungen (ILD-Boards) an Kliniken in Deutschland

National survey on the presence of interstitial lung disease multidisciplinary meetings at hospitals in Germany

Claus-Jürgen Bauer[1], Dirk Skowasch[2], Michael Kreuter[3], Okka W Hamer[4], Jürgen Behr[5,6], Sven Gläser[7], Claus Peter Heussel[8,9,10], Daniel Kütting[11], Andreas Krause[12], Gabriela Leuschner[5,6], Philipp Markart[13,14], Simon Michael Petzinna[1], Markus Polke[15], Valentin Sebastian Schäfer[1]

Elektronisches Zusatzmaterial:

Liste der deutschsprachigen Fragen

Frage 1. [Single-Choice]
Geben Sie bitte Ihre aktuelle berufliche Position an.
☐ Chefarzt/Chefärztin
☐ Oberarzt/Oberärztin
☐ Facharzt/Fachärztin
☐ Assistenzarzt/Assistenzärztin

Frage 2. [Freitexteingabe-Feld]
Geben Sie bitte Ihre Postleitzahl an.

Frage 3. [Single-Choice]
An welcher Einrichtung arbeiten Sie aktuell?
☐ Universitätsklinikum
☐ Nicht-universitäres Krankenhaus der Maximalversorgung
☐ Krankenhaus der Regelversorgung
☐ Spezialisiertes Krankenhaus (bitte angeben): [Freitexteingabe-Feld]

Frage 4. [Multi-Choice]
Welche Fachdisziplinen sind in der Fallbesprechung zu interstitiellen Lungenerkrankungen (im folgenden „ILD-Board“ genannt) in Ihrer Klinik vertreten? (Mehrfachantwort möglich)
☐ Pneumologie
☐ Radiologie
☐ Pathologie
☐ Rheumatologie
☐ Dermatologie
☐ Thoraxchirurgie
☐ Sonstiges (bitte angeben): [Freitexteingabe-Feld]

Frage 5. [Single-Choice]
Gibt es eine Mindestpräsenz von Fachdisziplinen, welche für das Zustandekommen des ILD-Boards an Ihrer Klinik vertreten sein müssen? (Wenn ja, bitte Fachdisziplinen benennen)
☐ Nein

☐ Ja (bitte angeben): [Freitexteingabe]

Frage 6. [Single-Choice]
Gibt es Fachdisziplinen, aus denen die Präsenz mehrerer Vertreter erforderlich ist (beispielsweise 'mindestens zwei PneumologInnen')?
☐ Nein
☐ Ja (bitte angeben): [Freitexteingabe-Feld]

Frage 7. [Single-Choice]
Das ILD-Board findet an Ihrer Klinik…
☐ in Präsenz statt
☐ virtuell statt
☐ beides (hybrid)

Frage 8. [Single-Choice]
Wie häufig findet das ILD-Board im Monat an Ihrer Klinik statt?
☐ Einmal/Monat
☐ Zweimal/Monat
☐ Dreimal/Monat
☐ Viermal/Monat bzw. wöchentlich
☐ Unregelmäßig, in Abhängigkeit von der Anzahl der Fälle
☐ Sonstiges (bitte angeben): [Freitexteingabe-Feld]

Frage 9. [Single-Choice]
Wie lange dauert im Durchschnitt eine gesamte ILD-Board-Sitzung an Ihrer Klinik?
☐ <15 Minuten
☐ 15-30 Minuten
☐ 30-60 Minuten
☐ 60-90 Minuten
☐ >90 Minuten

Frage 10. [Single-Choice]
Wie viele Fälle werden im Durchschnitt pro ILD-Board-Sitzung an Ihrer Klinik besprochen?
☐ 1-5 Fälle
☐ 5-10 Fälle
☐ 10-15 Fälle
☐ 15-20 Fälle
☐ 20-30 Fälle
☐ >30 Fälle

Frage 11. [Single-Choice]
Können auch externe Patienten von niedergelassenen Kollegen beziehungsweise auswärtigen Krankenhäusern für das ILD-Board angemeldet werden?
☐ Ja
☐ Nein

Frage 12. [Slider-Item: Zahlenwerte 0-100]
Wie viel Prozent der vorgestellten Patientenfälle in Ihrem ILD-Board stammen von externen Anmeldern?

Frage 13. [Single-Choice]
Existiert zur Anmeldung für das ILD-Board ein standardisiertes Formular?
☐ Ja

☐ Nein

Frage 14. [Multi-Choice]
Welche Informationen/Werte werden zur ILD-Boardanmeldung in diesem Formular abgefragt? (Mehrfachantwort möglich)
☐ Raucherstatus
☐ Symptomdauer
☐ Berufsanamnese
☐ Medikamentenanamnese
☐ Körperliche Untersuchung
☐ CRP-Wert
☐ Autoimmundiagnostik
☐ Bodyplethysmographie
☐ Bodyplethysmographie und Diffusion
☐ Befund der letzten CT und CT-Muster
☐ Werte der Bronchoalveolären Lavage
☐ Pathologiebefund
☐ Spezifische IgGs für exogen-allergische Alveolitis

Frage 15. [Single-Choice]
Beschränkt sich die Evaluation der radiologischen Befunde im Rahmen des ILD-Boards an Ihrer Klinik lediglich auf das Vorlesen des schriftlichen Befundberichts oder wird der radiologische Befund typischerweise auch bilddemonstriert und im ILD-Board das pulmonale Muster diskutiert?
☐ Typischerweise nur Vorlesen des schriftlichen Radiologiebefundes
☐ Typischerweise radiologische Bilddemonstration und Diskussion des pulmonalen Musters im ILD-Board

Frage 16. [Single-Choice]
Beschränkt sich die Evaluation der histopathologischen Befunde im Rahmen des ILD-Boards an Ihrer Klinik lediglich auf das Vorlesen des schriftlichen Befundberichts oder wird der histopathologische Befund typischerweise auch bilddemonstriert und diskutiert?
☐ Typischerweise nur Vorlesen des schriftlichen Histopathologie-Befundes
☐ Typischerweise histopathologische Befunddemonstration und Diskussion

Frage 17. [Multi-Choice]
Im Rahmen des ILD-Boards erfolgt an Ihrer Klinik… (Mehrfachantwort möglich)
☐ Das Stellen und Zuordnen einer spezifischen Diagnose
☐ Eine Empfehlung zur weiteren Diagnostik
☐ Eine Therapieempfehlung
☐ Die Erstellung eines standardisierten Ergebnisprotokolls

Frage 18. [Single-Choice]
Folgen Sie und Ihr interdisziplinäres Team in der ILD-Diagnostik typischerweise den Empfehlungen der „S1- Leitlinie interdisziplinäre Diagnostik interstitieller Lungenerkrankungen im Erwachsenenalter"?
☐ Ja
☐ Nein

Frage 19. [Slider-Item: Zahlenwerte 0-100]
Mit welchem Ergebnis/welcher Empfehlung ist die Besprechung eines Patientenfalls im ILD-Board typischerweise an Ihrer Klinik verknüpft? Geben Sie bitte eine Prozentzahl von 0-100 % an.

Empfehlung weiterer Diagnostik (z.B. Kryobiopsie) in _________% der Fälle

Frage 20. [Slider-Item: Zahlenwerte 0-100]
Mit welchem Ergebnis/welcher Empfehlung ist die Besprechung eines Patientenfalls im ILD-Board typischerweise an Ihrer Klinik verknüpft? Geben Sie bitte eine Prozentzahl von 0-100 % an.

Empfehlung einer rheumatologischen Vorstellung in _________% der Fälle

Frage 21. [Slider-Item: Zahlenwerte 0-100]
Mit welchem Ergebnis/welcher Empfehlung ist die Besprechung eines Patientenfalls im ILD-Board typischerweise an Ihrer Klinik verknüpft? Geben Sie bitte eine Prozentzahl von 0-100 % an.

Computertomographie-Wiederholung in _________% der Fälle

Frage 22. [Slider-Item: Zahlenwerte 0-100]
Mit welchem Ergebnis/welcher Empfehlung ist die Besprechung eines Patientenfalls im ILD-Board typischerweise an Ihrer Klinik verknüpft? Geben Sie bitte eine Prozentzahl von 0-100 % an.

Therapieempfehlung in _________% der Fälle

Frage 23. [Slider-Item: Zahlenwerte 0-100]
Mit welchem Ergebnis/welcher Empfehlung ist die Besprechung eines Patientenfalls im ILD-Board typischerweise an Ihrer Klinik verknüpft? Geben Sie bitte eine Prozentzahl von 0-100 % an.

Gesicherte Diagnose in _________% der Fälle

Frage 24. [Single-Choice]
Finden im ILD-Board an Ihrer Klinik Patientenwiedervorstellungen zur Verlaufsbesprechung (bspw. nach erfolgter Kryobiopsie oder bei klinischer Verschlechterung) statt?
☐ Nein
☐ Sonstiges (bitte angeben): [Freitexteingabe-Feld]

Frage 25. [Slider-Item: Zahlenwerte 0-100]
In wie viel Prozent der Fälle muss die Besprechung des Patienten im ILD-Board aufgrund fehlender Befunde verschoben werden?

Frage 26. [Freitexteingabe-Feld]
Wie könnte man zukünftige ILD-Boards weiter verbessern?
